# Eliminating Anisotropy of 7085 Alloy Forgings via Temperature Combination Control During Two-Stage Multi-Directional Forging

**DOI:** 10.3390/ma18020391

**Published:** 2025-01-16

**Authors:** Xiao Yin, Wensheng Liu, Xin Tan, Mingdong Wu, Shuo Yuan, Daihong Xiao, Lanping Huang

**Affiliations:** National Key Laboratory of Science and Technology on High-Strength Structural Materials, Central South University, Changsha 410083, China; 213301042@csu.edu.cn (X.Y.); liuwensheng@csu.edu.cn (W.L.);

**Keywords:** Al-Zn-Mg-Cu alloy, multi-directional forging, anisotropy, uniformity, microstructures evolution, recrystallization

## Abstract

Due to its high mechanical properties and low quench sensitivity, 7085 aluminum alloy is suitable for the aircraft industry. However, large cross-section forgings of 7085 alloy usually have over 40% anisotropy in mechanical behaviors, especially in the vertical direction. In this study, two-stage multi-directional forgings (MDFs) with different temperature combinations, isothermal medium-temperature composite MDF (MC-MDF) and isothermal hot MDF (H-MDF), were applied to 7085 aluminum alloy ingots. The results indicate that MC-MDF achieved anisotropy below 10% without losing ultimate tensile strength (UTS). Three-dimensional (3D) microstructure analysis suggested that the MC-MDF samples accumulated higher dislocation density and exhibited an enhanced recrystallization structure. The elongation of the vertical direction increased significantly, which lowered the directionality of MC-MDF and increased the effective utilization rate of forgings. Also, MC-MDF obtained a lower yield strength (YS) due to the forging temperature in exchange for higher work hardening and a ductility increase. The average 3D UTS, YS, and EL values of MC-MDF are 554 MPa, 472 MPa, and 13.4%, and the index value reflecting the anisotropy of EL decreased from 14.0% to 8.6% for H-MDF.

## 1. Introduction

Al-Zn-Mg-Cu alloys have been widely used in the aircraft, aerospace, transportation, and defense industries due to their low density, high specific strength, high fracture toughness, good workability, and reasonable stress corrosion cracking resistance [1,2,3]. In particular, 7085 aluminum alloys have much lower quench sensitivity when compared to other Al-Zn-Mg-Cu alloys, such as 7075, 7050, 7055, 7020, and so on [4,5], which supports its forging applications in airplanes like the C919, Su-30, and Airbus A380 [6]. With the development of industries, the demand for large-scale, low-anisotropy, and high-mechanical-performance 7085 alloys is becoming urgent. However, the traditional 7085-T7451 thick plate shows an anisotropy value of more than 40% for the elongation property between the height and the other two directions at a thickness between 150 and 175 mm [7]. The high anisotropy of the 7085 forgings greatly restricts its industrial application, causing security and economic problems.

The anisotropy of aluminum alloys is affected by a variety of factors, including heat treatment (precipitating phases), crystal orientation, microstructure, and external reinforcing agents. A study by Hu et al. [8] showed that the anisotropy of 7050 aluminum alloys was significantly different under different heat treatment conditions, especially under the condition of overage. Through Schmid factor analysis, their study revealed that crystal orientation is a key factor in the formation of anisotropy and is highly related to heat treatment conditions.

In a study on the effect of plastic anisotropy on the ductility of materials, Frodal et al. [9] used nonlinear finite element simulation tensile tests with different orientations and found that low yield strength and high work hardening can significantly improve the ductility of aluminum alloys. Bhattacharyya et al. [10] verified the important influence of precipitation particle distribution on the plastic anisotropy of 7085 aluminum alloy through a model that predicts the effect of precipitation-induced reaction forces. With deeper aging, the alloy becomes more isotropic.

The experimental data of Khadyko et al. [11] further proved the dominance of the crystal orientation of AA6063 aluminum alloy in plastic anisotropy. The results obtained through the analysis of electron backscatter diffraction (EBSD) show that heat treatment has little effect on anisotropy, providing strong evidence for understanding the dominant role of crystal structure on material properties based on the difference between the 0° and 45° directions. Park et al. [12] pointed out that the differences in microstructure will cause significant differences in the mechanical behavior of materials in different directions by testing the microstructure and mechanical properties of AA2024-T4 aluminum alloy extruded bars. Anderson et al. [13] combined digital image correlation technology and the VPSC model to verify the influence of Piobert–Lüders bands on anisotropy characteristics in AA3104 alloy and provided an in-depth analysis at the microscopic level. Li et al. [14] studied the anisotropy of Al-Zn-Mg-Sc-Zr alloys, revealed the significant effects of different angles on the strength and ductility of materials through the Schmid factor and mechanical property data, and pointed out the close relationship between anisotropy and material microstructure.

Finally, Li et al. [15] studied the anisotropy characteristics of TiB2-reinforced 2024 aluminum matrix composites and found that the distribution of nanoparticles in the materials can significantly improve the isotropy of the materials by promoting the recrystallization process. In summary, the studies above provide data support and a theoretical basis for understanding the causes of anisotropy in aluminum alloys. Among factors such as different microstructures, heat treatment states, and external reinforcing agents, different microstructures are believed to be the most important factor that affects the anisotropy of aluminum alloy. During material production, the stage that affects the microstructure the most is the deformation process.

Among different deformation techniques, multi-directional forging (MDF) applies compression and stretch repeatedly on three different axes between different passes of forging without the help of special dies, which makes it simple, cost-effective, low-anisotropy, and suitable for large bulk materials when applied in industries [16,17,18,19,20]. However, with traditional MDF, it is still hard to decrease the anisotropy of 7085 forgings to a level under 10%, and in industrial products, the anisotropy usually exceeds 40% [19].

Many researchers have studied how to decrease anisotropy via MDF. Tang et al. [21] reported that PT3 forging pretreatment with the MDF process enhances the dissolution of non-equilibrium phases and accelerates the recrystallization behavior of alloys. The average grain size decreased from 356 µm to 186 µm, tensile strength increased by 20–30 MPa for all three directions, and the elongation of height direction also increased to 6.3%, which reduced the anisotropy of Al-Cu-Li alloys. He et al. [22] studied the effects of forging temperature on the microstructure and mechanical properties of MDFed Al-Cu-Li alloys. The results showed that average grain size increased with a decrease in forging temperature due to more significant static recrystallization (SRX). Given the stronger SRX, the average grain size became larger, and the grain structure became more uniform and equiaxed; the elongation of samples was maintained with decreased anisotropy in three dimensions of the forgings. Jamali et al. [17] studied the anisotropy and mechanical properties of an extruded MDFed ZK60 Mg alloy. This resulted in a low anisotropy, fine-grained, and homogeneous microstructure achieved after nine passes of MDF in all three dimensions. Fully recrystallized microstructures, after nine passes, have homogenized the texture and grain sizes, eliminating the differences between the dimensions of the alloy. Wang et al. [23] studied the mechanical properties and texture evolution of large-scale MDFed Mg-Gd-Y-Zn-Zr-Ag alloys, increasing the number of passes from 9 to 27. The results showed that the anisotropy increased from 2.5% to 10.9% with an increase in mechanical properties. High passes enhanced tensile properties along the FD due to significant effects from dynamic precipitation and dynamic recrystallization. The above studies provide multiple methods, such as forging temperature, pass, accumulative strain, and pretreatment, for affecting the anisotropy of forgings. The key to linking these factors with the anisotropy of alloys is controlling the grain structure and texture via deformation and recrystallization during MDF. However, previous studies usually focus on mechanical properties and how different factors affect anisotropy; optimization, which focuses on decreasing 3D anisotropy for industrial production, is rare. To minimize anisotropy, a significant loss of mechanical strength has been widely observed in current studies, which decreases their application value. Thus, developing a feasible MDF process to achieve high mechanical properties with low anisotropy is meaningful for industry.

In this study, a two-stage MDF with inter-annealing was designed with different combinations of forging temperatures to develop forgings with low anisotropy and high mechanical properties. Current studies usually focus on single-parameter controlling during the MDF process, such as forging temperature, the number of passes, strains per pass, different types of die, and so on. For easier and more precise control, the forging process is usually designed to be short and simple. However, the two-stage MDF applied in this work aimed to combine both advantages from different forging temperatures and construct highly comprehensive mechanical properties with low anisotropy on 7085 forgings. The two-stage forging with inter-annealing between stages was applied to release strain energy uniform property and enable more forging passes. Meanwhile, different forging temperature combinations were applied to determine the cooperative control of deformation areas, microstructures, and precipitation during aging. Comparing the advantages and weaknesses, it is worthy to extend MDF for better properties. Through multi-scale observation, it was found that the fraction of recrystallized structures, dislocation density, work hardening, average grain size, precipitation, and mechanical properties were affected by different temperature combinations during MDFs. Anisotropy of the 7085 forging was nearly eliminated with an optimized forging temperature combination. Multi-scale characterizations were applied on three dimensions (3Ds) of the specimen; the microstructural evolution, corresponding mechanisms, and anisotropic behaviors of 7085 forgings are also investigated and discussed in detail.

## 2. Experiment

In this work, the experimental material was obtained from a commercial 7085 (Chinalco Southwest Aluminium, Chongqing, China) aluminum ingot (Al-7.28Zn-1.49Mg-1.68Cu-0.11Zr, wt.%). The size and shape of both samples were the same, measuring 100 mm × 100 mm × 100 mm. To match real industrial productions, both samples were cut from the center area of the pre-homogenized ingot and subjected to MDFs with the same forging steps but different forging temperature combinations.

**Figure 1:**
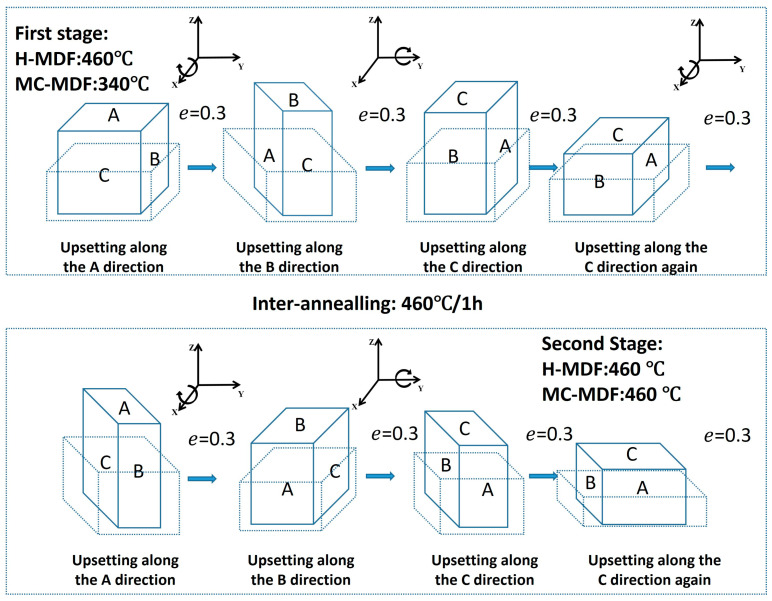
Schematics of H-MDF and MC-MDFt.

The experiments were carried out via a 3 MN hydraulic press with speed of 5 mm/s. Figure 1 presents the schematic diagram of the different MDF processes (the pre-deformed forging is represented by a blue solid line, while the deformed forging is represented by a blue dotted line). All samples were defined as A, B, or C faces and endured 8 pass compressions in total during the two stages of the MDF process. During the first stage, each sample was compressed on the A face first and then turned to the B face, and the height of the forging was reduced by 30%, where the true strain value was around 0.26 on the compressed face. After the first two passes, compression was applied to the C face twice in a row using the same true strain as before each time. The total true strain of the MDF process is 2.08. Between the first and second stages of forging, a one-hour annealing process at 460 °C was conducted, which can help balance the release of strain energy and while keeping the effects of forging. As the inter-annealing temperature matches the forging temperature of the second stage for both MDFs, the efficiency of the two-stage MDFs increases as well. The second stage of forging was a repetition of the first stage on each pass. After all passes were performed, the samples were air-cooled, and the height of face C for both samples was compressed to around 50% of its original height. The final molding size was around 150 mm × 120 mm × 50 mm, and the total strain was 2.4 for both samples. For isothermal hot MDF (H-MDF), in this study, the starting temperature for both the first stage and the second stage of the forging process was 460 °C. The isothermal medium temperature composite MDF(MC-MDF) was started at 340 °C in the first stage, while that of the second stage was started at 460 °C.

After MDFs, heat treatments applied to both samples were the same and are given as follows: the solution was treated at 470 °C for 2 h, water quenched, and then subjected to T74 aging (aged at 120 °C for 7 h, switched to 160 °C for 10 h, and finally, water quenched). We used chamber electric furnaces for forging heating, annealing, and the solid solution, while the aging process was applied using the aging furnace. The whole MDF process and control of temperature are easily reproducible in industrial production.

The microstructures were characterized by scanning electron microscopy (SEM: OXFORD-X-Max20, ZEISS, Jena, Germany), optical microscope (OM: RX50M, Sunny Optical Technology, Yuyao, China), and transmission electron microscopy (TEM: Talos F200X, Thermo Fisher Scientific, Waltham, MA, USA). High-angle annular dark-field (HAADF) images were applied to characterize the morphology of grain boundary precipitations (GBPs). The center of the specimens, which are parallel to each plan, are cut down for microstructural analysis. The evolution of second-phase particles and fracture characteristics were examined via SEM. All specimens were prepared by grinding and mechanical polishing. The grain structure characteristics of samples from the ingots after forging and aging were obtained using electron backscattering diffraction (EBSD: NordlysMax2, Oxford Instruments, Oxford, UK). The obtained information and images were analyzed by AztecCrystal software (Version 2.1, Oxford Instruments, Oxford, UK). All EBSD samples were prepared through electrolytic polishing with an electrolyte mix of 10% HClO4 and 90% CH_3_OH. A hydraulically driven universal testing machine (Instron 3699, Instron, Norwood, MA, USA) was applied for tensile testing, where the tensile rate was 2 mm/min. The uniformity of the forgings was tested through a comprehensive measurement of the tensile properties. Tensile samples were obtained from the center of all three directions: (length (LT), width (TL), and height (ST)). For each sampling position, three tensile samples were processed. The average values of tested results were used as the final test values to minimize errors. The aging-treated samples were thinned to around 80 µm via grinding. Then, the samples were punched into 3 mm diameter disks and further thinned by an electro-polishing device. Final polished samples were observed using a transmission electron microscope (TEM).

**Figure 2:**
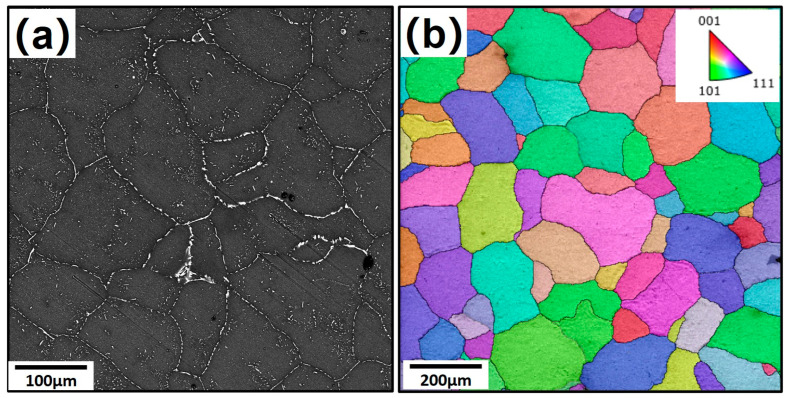
Initial state of the homogenized 7085 ingot: (**a**) SEM image; (**b**) EBSD image.

Figure 2 presents the initial microstructures of homogenized 7085 aluminum alloy ingots. From Figure 2a, it can be seen that a large amount of primary phase particles are still segregated on grain boundaries in the ingot, mainly found in the forms of AlZnMgCu and Al_7_Cu_2_Fe. The statistical analysis of Figure 2b indicates that the average grain size of the initial structure is 103.9 µm in diameter of the equivalent circle.

## 3. Results

### 3.1. Three-Dimensional Microstructures

**Figure 3:**
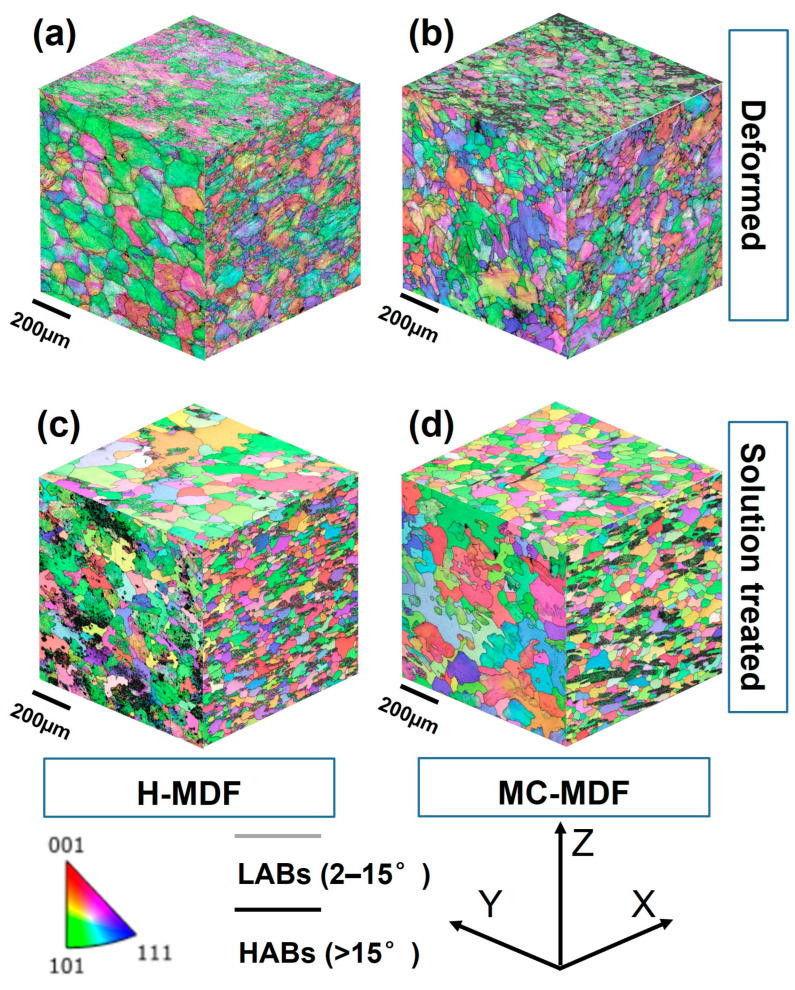
Three-dimensional IPF images presenting specimens after being forged and solution-treated via (**a**,**c**) H-MDF; (**b**,**d**) MC-MDF; (**a**,**b**) forged state; and (**c**,**d**) the solution-treated state.

Forgings underwent different MDFs, which resulted in different grain structure morphologies and mechanical performances. Figure 3 presents 3D EBSD images that correspond to the grain structures of the 3Ds (length, width, and height) of forgings after forging and solution treatment. Grains of H-MDF were much larger in size than that of MC-MDF. Thus, the corresponding deformation degree was relatively smaller, with a higher tendency of dynamic recovery, while that of MC-MDF had finer recrystallized grains surrounding larger deformed grains after forging.

In Figure 3c,d, inverse pole figures (IPFs) on three dimensions of different MDF samples after heat treatment are presented. The orientation of the grain structures of each forging had significantly changed after solid solution and aging, and the average grain size had been greatly improved based on grain growth. Grain boundary characteristics of forgings were inherited from the deformed morphology, with a sharp decrease in the proportion of LABs. After aging, the grain structure of the H-MDF showed thin, flat, and low-toughness grain morphologies. Long grain boundaries are perpendicular to the height direction, which easily becomes a crack extension path when tensile force is applied. The grain morphology characteristics of MC-MDF are more uniform in 3D than that of H-MDF with lower directionality, which contains many large, recrystallized grains mixed with smaller sub-grain structures.

### 3.2. Three-Dimensional Mechanical Properties

**Figure 4:**
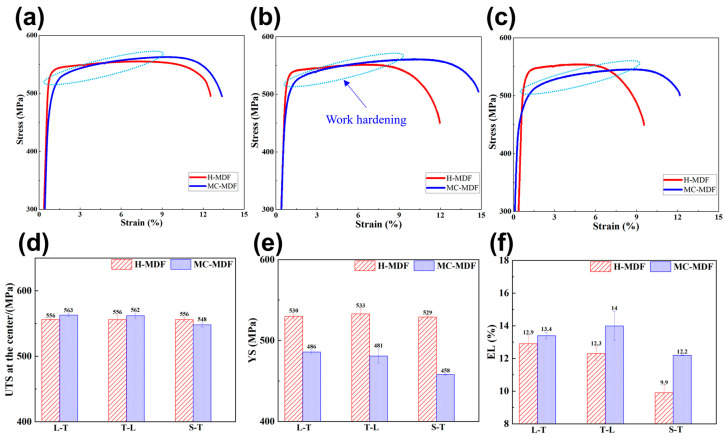
Stress–strain curves and mechanical properties of H-MDF and MC-MDF samples at the 3D direction after T74 aging, (**a**) curves on L-T, (**b**) curves on T-L, (**c**) curves on S-T, (**d**) UTS, (**e**) YS, and (**f**) EL.

After MDFs, significant differences in mechanical properties between forgings were found in addition to properties of 3D directions. Figure 4 and Table 1 present 3D mechanical properties of different MDF samples, namely the ultimate tensile strength (UTS), yield strength (YS), and elongation (EL) of the forging center at length (L-T), width (T-L), and height (S-T) directions. In terms of strength performance, the average 3D UTS of H-MDF and MC-MDF were 556 MPa and 558 MPa, and the average 3D YS were 531 MPa and 475 MPa. The UTS difference between H-MDF and MC-MDF is ignorable, but that of YS is obvious. From the perspective of elongation, the average EL of H-MDF and MC-MDF samples were 11.7% and 13.5%, respectively. MC-MDF had much higher EL in the S-T direction than that of H-MDF.

An index of anisotropy (IDA) is applied to represent the anisotropic behaviors of mechanical properties [24]; the larger the IDA value is, the higher the anisotropy will be.

(1) $$IDA=\frac{2X_{max}-X_{min}-X_{mid}}{2X_{max}}\times 100\%$$

where $X_{max}$ represents the max values, $X_{min}$ represents the minimum value, and $X_{mid}$ represents the median value of the properties. Based on the properties of Figure 4 and Table 1, calculated IDA values of forgings are presented in Table 2. The maximum value of IDA among UTS and YS is 3.4%, which indicates that the anisotropic behavior of strength can be ignorable. However, when it comes to IDA values of EL, samples of MC-MDF had much lower IDA values than those of H-MDF. The IDA on EL of MC-MDF is 8.6%, while that of H-MDF was nearly 50% higher, at 14.0%. The main difference comes from EL on the height direction of H-MDF, which is lower than the other two directions, and increased values of IDA. For L-T and T-L directions, the average EL increase rate from H-MDF to MC-MDF is 8.7%. However, in the S-T direction, the increase rate from H-MDF to MC-MDF rose to 21.2%. MC-MDF increases EL on all dimensions and shows stronger effects on the height direction of the forging.

Compared to the 3D mechanical properties of different forgings, it was found that MC-MDF samples had lower YS but increased EL for every direction, especially in height direction when compared to H-MDF with much more obvious strain hardening effects. Also, samples of MC-MDF samples showed less difference in 3D mechanical properties based on calculated IDA. Thus, with the highest mechanical properties difference, the latter detection parts are more concentrated on microstructures of forgings height (S-T) direction.

**Figure 5:**
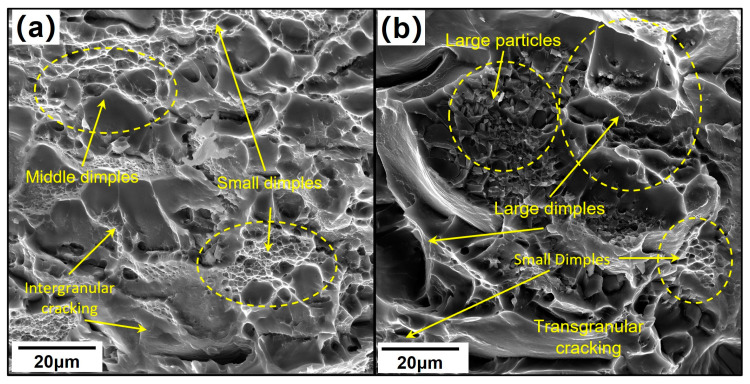
SEM images of tensile fracture surfaces at T74 aged samples: (**a**) H-MDF; (**b**) MC-MDF.

SEM images in Figure 5 present a tensile fracture surface on the S-T direction of forgings. The fracture surface of H-MDF samples has many small and middle-size dimples, while dimples of MC-MDF have a higher size distribution. From H-MDF to MC-MDF, the tensile fracture characteristics changed from an intergranular fracture mixed trans-granular fracture to a mainly trans-granular fracture. The MC-MDF fracture surface has more and finer dimples with the existence of large rock sugar-like phase particles, which indicated that ductile trans-granular fractures dominated fracture mode.

### 3.3. Microstructures Under Different States

**Figure 6:**
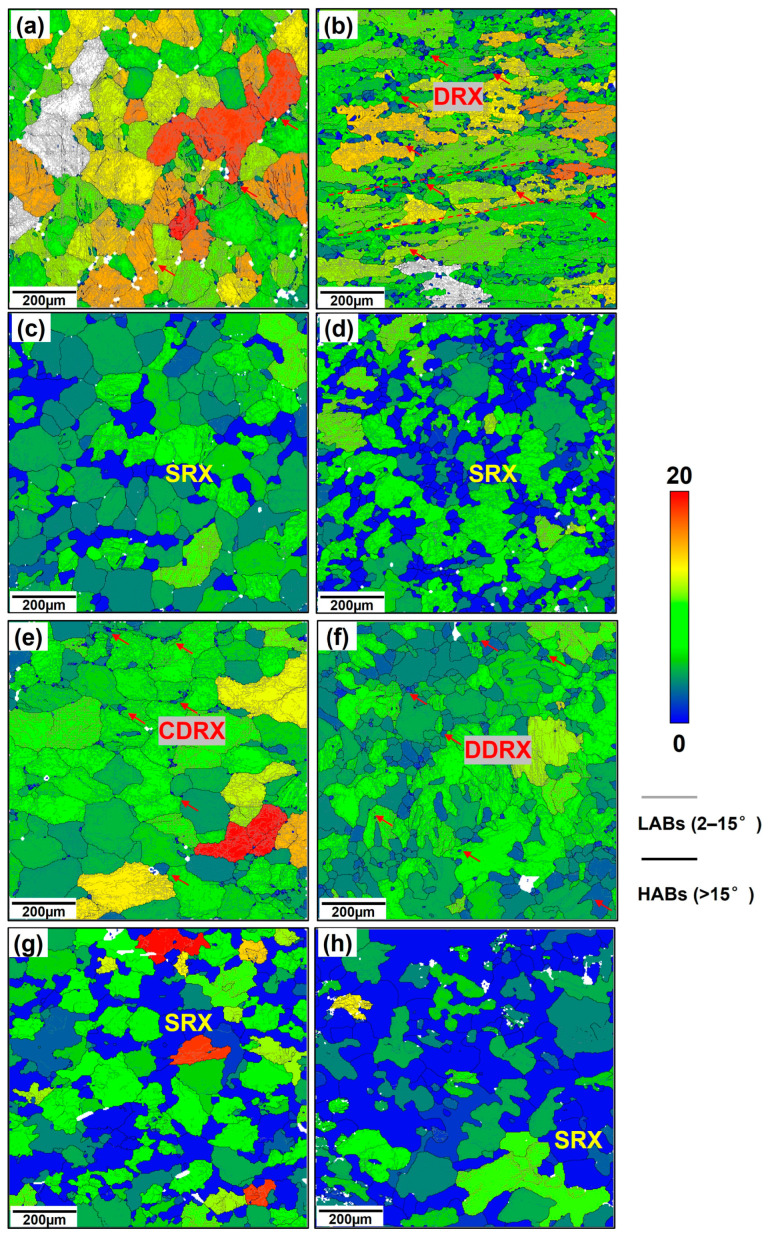
The GOS maps of forgings under different states: (**a**,**c**,**e**,**g**) H-MDF; (**b**,**d**,**f**,**h**) MC-MDF; (**a**,**b**) after the first stage; (**c**,**d**) inter-annealing state; (**e**,**f**) after the second stage; (**g**,**h**) solution-treated. Blue areas represent recrystallized structures (<2°); green and yellow areas represent substructures (2–15°); red and no-color areas represent deformed structures (>15°); GOS map and grain size distribution of different forgings under different states. Red arrows represent DRX.

**Figure 7:**
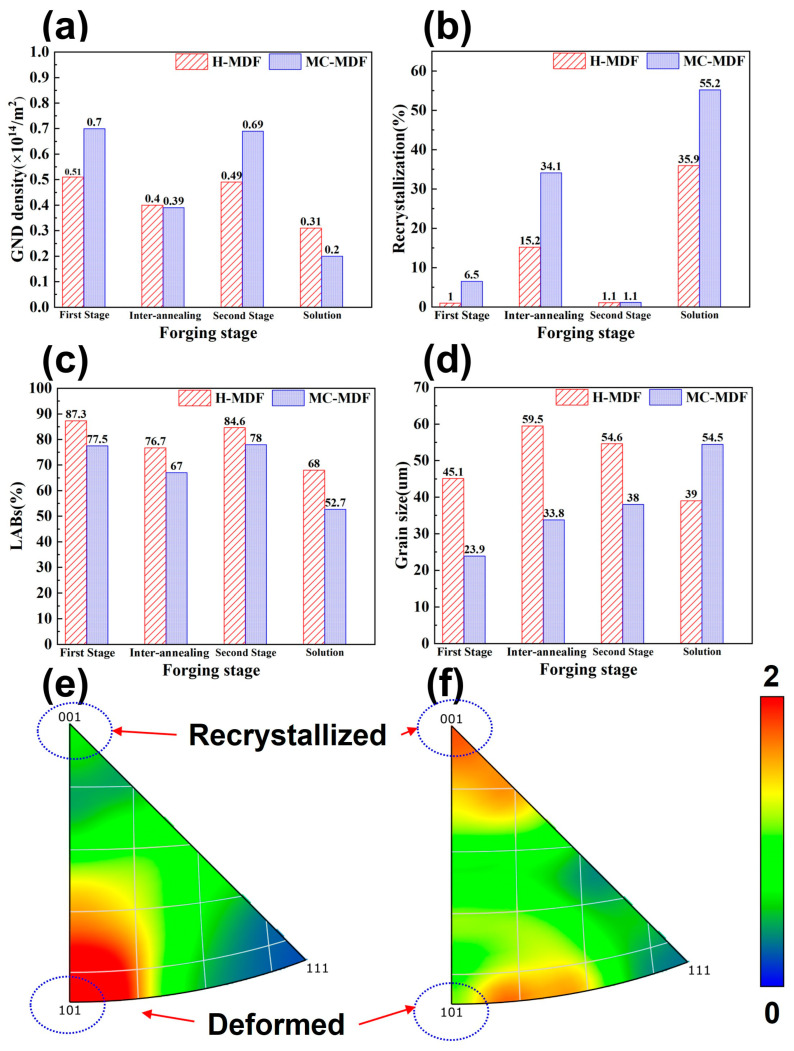
Comparison between (**a**) GND, (**b**) recrystallization, (**c**) LABs, (**d**) grain size and IPF between H-MDF and MC-MDF forgings under different states: (**e**) IPF//Z0 of H-MDF and (**f**) IPF//Z0 of MC-MDF.

After the first stage, forging of MDF, the grain structure at the center of forgings at height directions presented different morphologies. Figure 6a–f and Figure 7a–d illustrate grain boundaries distribution and grain orientation spread (GOS) of forged forgings. Usually, grain boundaries with orientation differences larger than 15° were identified as high-angle grain boundaries (HABs), which were formed by recrystallization or from original deformed grains, while low-angle grain boundaries (LABs) were identified as grain boundaries with 2–15° orientation differences (formed by sub-grain boundaries). For grain structures, an orientation spread value lower than 2° was defined as recrystallized structures, 2–15° as sub-structures, and >15° as deformed structures. During the first forging stage, the deformation temperature of MC-MDF was 340 °C, while that of H-MDF was 460 °C. During the first stage of forging, the grain size of H-MDF and MC-MDF dropped from 103.9 µm to 45.1 µm (H-MDF) and 23.9 µm (MC-MDF), respectively. The H-MDF sample exhibited a larger grain size than the MC-MDF, which exhibited a higher DXR degree (6.5%). This high degree produced numerous new recrystallized grains, leading to a decrease in the average grain size.

Inter-annealing between the two forging stages included heating at 460 °C for 1 h. From Figure 6c,d and Figure 7d, the grain size of H-MDF and MC-MDF changed to 59.5 µm and 33.8 µm, respectively, indicating different trends in states after the first stage of forging. The growth and drop in grain size in H-MDF and MC-MDF were due to their energy difference, which is controlled by dynamic recrystallization behaviors. The percentage of recrystallization structures (RX) of H-MDF and MC-MDF changed from 1.0% (H-MDF) and 6.5% (MC-MDF) to 15.2% (H-MDF) and 34.1% (MC-MDF). During annealing, the statistic recrystallization increasing from a deformed to an annealed state of H-MDF is 14.2%, and that of MC-MDF nearly doubled to 27.6%. Samples of MC-MDF present both a higher recrystallization degree and a larger increase rate than H-MDF.

Figure 6g,h contain the evolution and distribution of grain boundaries, grain sizes, and GOS from the forged state to the solution-treated state in the S-T direction of different samples. The forged H-MDF samples have a higher proportion of LABs (84.6%) and a larger average grain size (54.6 μm) but a lower fraction of recrystallized grains (1.1%). For MC-MDF, the LABs fraction was 78.0%, and the average grain size was 38.0 µm. The proportion of recrystallized grains in MC-MDF is 1.1% after the second forging stage at the same temperature. For both the H-MDF and MC-MDF process, DRX is the dominant recrystallization nucleation mechanism, which produces many new recrystallized grains and leads to a decrease in the proportion of LABs.

Strain energy distribution can be presented through data of geometric dislocation density (GND) in Figure 7a. The average dislocation densities are in direct proportion to strain energy. The GND of MC-MDF is 0.69 × 10^14^/m^2^, higher than that of H-MDF (0.49 × 10^14^/m^2^).

After the solid solution, many substructures transformed into recrystallized structures, and the proportion of high grain orientation diffraction areas was greatly reduced. The proportion of recrystallized structures followed the trends in strain energy under deformed states. The proportion of recrystallized structure in the H-MDF sample is 35.9% with a high average geometric dislocation density of 0.31 × 10^14^/m^2^, while that of MC-MDF are 54.2% and 0.20 × 10^14^/m^2^, respectively. From the deformed state to the aging state, the geometric dislocation densities of forging were reversed, which indicated that MC-MDF released more strain energy during heat treatment and obtained a higher degree of RX.

The proportion of LABs decreased, and the average size of the grain increased after heat treatment for both forging. The trends in the proportion of LABs followed the order of GND for solution-treated forgings. Consistent with the trend in a deformed state, H-MDF (Figure 7c) still has the highest proportion of LABs with a small drop from 84.6% to 68.0% and its average grain size reducing to 39.0 µm. By contrast, the proportion of LABs of MC-MDF (Figure 7c) dropped from 78.0% to 52.7%, while its average grain size increased from 38.0 µm to 54.5 µm.

Based on IPFs in Figure 7, the H-MDF sample has a 110//RD texture tensity of 2.15, while the tensity of 001//RD texture is 0.64. Regarding the MC-MDF sample, the tensity of 110//RD decreases to 1.13 while that of 001//RD increases to 1.79. For FCC Al alloys, the 110 and 001 textures correspond to the deformed and recrystallized textures, respectively. The forging temperature combination of MC-MDF not only makes recrystallized grains become dominant but also weakens the deformed structure [25]. As the deformed structure contributed to isotropy, it indicates that a sample of MC-MDF has lower isotropy than H-MDF, which corresponds to mechanical properties and IDA values above.

**Figure 8:**
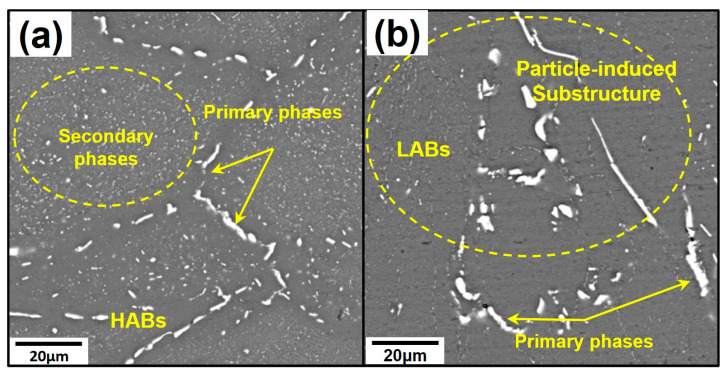
SEM images of different forgings after MDF: (**a**) H-MDF; (**b**) MC-MDF.

The SEM images in Figure 8 present the phase particle characteristics of forged state samples in the S-T direction. The coarse particles are primary phases like AlZnMgCu and Al7Cu2Fe, which have not been fully solidified into the matrix or are hard to dissolve due to their high melting point, and fine particles are secondary precipitates that reprecipitate during air cooling after forging [22]. ImageJ (version 1.8.0, National Institutes of Health, USA) mapping statistical analysis was applied to measure phase proportions under lower magnification images. The area fraction of secondary phases in H-MDF is 11.3%, which is higher than that of MC-MDF (3.4%). Meanwhile, remine primary phases in H-MDF and MC-MDF occupy 2.7% and 3.9% area fractions, respectively. H-MDF samples have a higher proportion of secondary phases but a lower proportion of primary phases under a deformed state upon their higher forging temperature.

The distribution of phases is influenced by forging processes. For H-MDF, secondary phases mainly existed inside grains, and agglomerated phases segregated on grain boundaries with a precipitation-free zone (PFZ). In samples of MC-MDF, the agglomerated phases are partially encapsulated in substructures with much fewer secondary phases existing.

### 3.4. Precipitation

**Figure 9:**
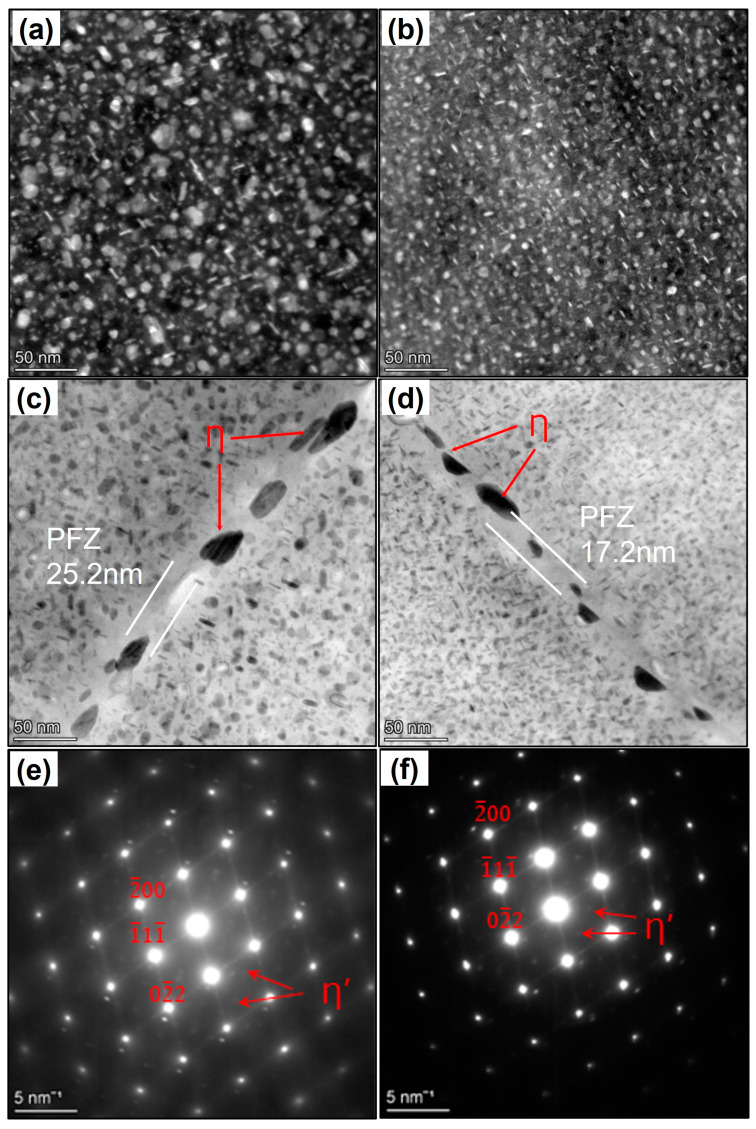
TEM images and corresponding SAED patterns of grain interiors after aging: (**a**,**c**,**e**) H-MDF; (**b**,**d**,**f**) MC-MDF; (**a**,**b**) precipitation in grains; (**c**,**d**) grain boundaries; (**e**,**f**) SAED patterns.

Figure 9 illustrates the TEM images of the forgings in the <011> _Al_ direction after being heated at 470 °C for 2 h and then T74 aging-treated at (120 °C/7 h+ 160 °C/10 h). All corresponding SAED patterns under different MDF processes were analyzed. The main precipitated phases in these 7085 alloys are the Al_3_Zr, η, and η’ phases. The plate-shaped η’ applied {111} of α-Al as the habit surface, and its orientation relationship with the matrix was (0001)_η’_//(111)_Al_, (1010)_η′_//(110)_Al_, and (1120)_η’_//(211)_Al_. Observed from the [1 1 0]_Al_ band axis, the two habitual planes of (-1 1 -1)_Al_ and (-1 -1 1)_Al_ of the η’ phase were parallel to the electron beam, and the T_1_ phases were the narrowest needle-like phases on the matrix plane. In the center of forging, Al_3_Zr (spherical), η (on grain boundary), and η’ (intra-granular) phases were denser in MC-MDF, while H-MDF had the larger average precipitate phase size. For MC-MDF, the average size of the precipitates (8.4 nm) was smaller, with a higher precipitate density than that of H-MDF (12.1 nm). When compared to the morphology of grain boundaries, H-MDF had a wider precipitates free zone (PFZ), which was 25.2 nm, and a larger grain boundary precipitation phase (41.8 nm in length). MC-MDF had the narrower PFZ (17.2 nm) with smaller grain boundary precipitation phases (GBPs), which were 23.8 nm in length.

## 4. Discussion

### 4.1. Evolution of Microstructure

Generally, the DRX behavior of aluminum alloy could be classified into discontinuous dynamic recrystallization (DDRX), which mainly occurs on GBs through bulging out to nucleate and growth [26], and continuous dynamic recrystallization (CDRX), which is usually encouraged by the rotation of sub-grains inside deformed grains [27]. During MDFs, DRX was achieved with a significant amount of thermal activation energy and strain energy, and CDRX could be tenser with relatively lower forging temperature and higher strain rate, while DDRX could dominate when higher temperatures and restrict strain energy [18]. The strain energy of forgings increased with decreasing forging temperature under the same forging passes and enhanced the merger, rotation, nucleation, and growth of dislocations and sub-grain structures. Strain energy difference caused by temperature difference during MDFs which resulted in different levels of DRX degree after forging and affected SRX degree after heat treatment. Long et al. [28] proved that in suitable temperature and pass ranges, the lower the forging temperature during MDF, the higher the CDRX tendency for Al-Zn-Mg-Cu alloys.

**Figure 10:**
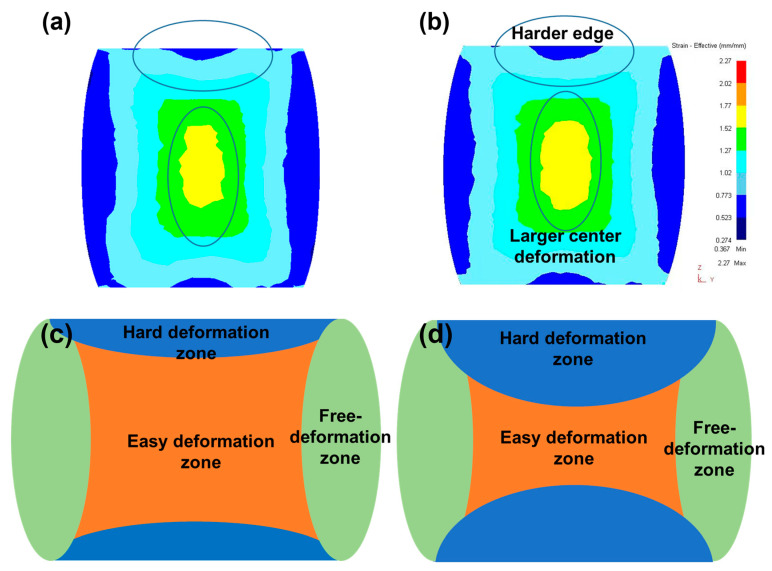
Schematic of three deformation zones and simulation of equivalent strain distribution in MDF process: (**a**,**c**) H-MDF; (**b**,**d**) MC-MDF; (**a**,**b**) simulation of equivalent strain distribution on forgings; (**c**,**d**) schematic of deformation zones.

Since the forging passes and accumulated strains of H-MDF samples and MC-MDF samples were the same, the difference in accumulated deformation storage energy on different forging samples was mainly caused by the difference in forging temperature. During MDFs, the free deformation zone and the hard deformation zone of the forgings alternated with the axial change, while the center of forging belonged to the easy deformation zone, as presented in Figure 10c,d [29]. The strain accumulation rate at the center is higher than in other areas, thus making the center area with the largest equivalent strain in forging. Figure 10a,b presents a simulation of the equivalent strain distribution on forgings after MDFs via Deform-3D (version 6.1, Scientific Forming Technologies Corporation, USA) software. It indicates that MC-MDF stored higher strain energy than H-MDF. The hard deformation zone is expended due to the hardening effect caused by lower forging temperature; thus, the deformation degree of easy deformation is even tenser.

**Figure 11:**
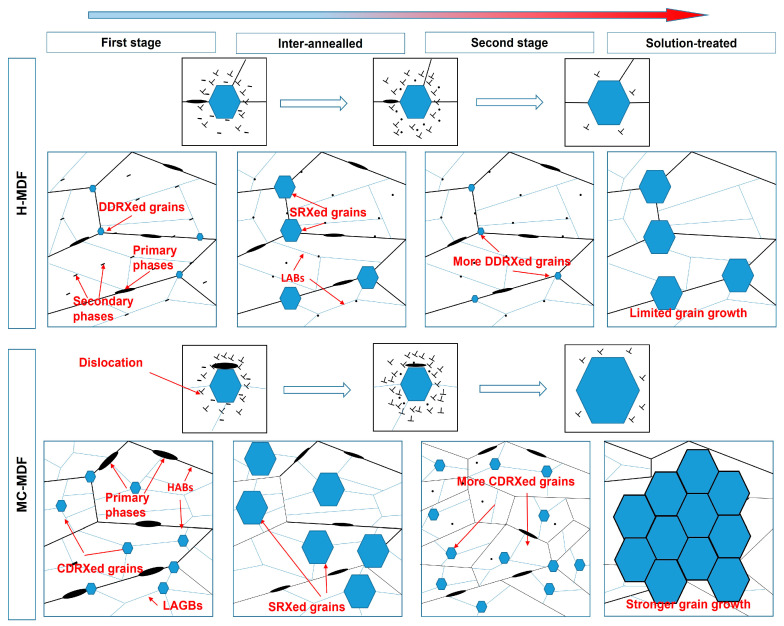
The schematic diagrams describe the microstructure evolution of H-MDF samples and MC-MDF samples.

The schematic diagram of grain structure evolution during MDF and heat treatment is shown in Figure 11. Through inter-annealing, partial strain energy was released, which caused the growth of recrystallized grains, and the proportion of LABs decreased for both forgings. MC-MDF sample has larger residual particles than that of H-MDF due to lower forging temperature in stage one. Recrystallized grains, together with residual primary phases and original GBs, became obstacles during the second stage of MDF passes and enhanced the DRX process [30]. MC-MDF samples had smaller grain sizes, higher recrystallization degrees, more primary phases, and a higher fraction of HABs than that of H-MDF, which made samples of MC-MDF harder to forge during the second stage. After the second stage of MDFs, MC-MDF had a lower proportion of LABs. The strain energy and temperature provided a driving force for recrystallization. The lower the forging temperature during MDF is, the stronger the work-hardening effects of forgings would be.

For deformed states, recrystallization structures are mainly obtained from DRX during the forging process. Recrystallized grains tended to nucleate around the intersection of grain boundaries or large crystalline phase particles with high strain energy due to the accumulation of dislocations and defects during forging and heat treatment processes [23,31]. The phenomenon that large crystalline phases were wrapped by substructures or recrystallized structures was observed widely in MC-MDF due to larger primary phases and higher strain energy stored during the first forging stage. H-MDF showed a lower DRX degree as fewer loads were required to deform forging at higher temperatures [27]. The new SRX grains, as well as the remaining primary phases, enhanced CDRX during the second stage of forging by obstructing grain boundary migration and supplying nucleation points [32]. H-MDF samples stored less strain energy than MC-MDF, and the driven force for SRX and high energy area during inter-annealing was less. Recrystallized grains tended to nucleate around the intersection of grain boundaries or large crystalline phase particles with high energy caused by the accumulation of dislocations and defects [33]. MC-MDF samples had more high energy areas for nucleation and resulted in more recrystallized grains. Also, both low forging temperature and intracrystalline phases lead to a higher trend in CDRX in MC-MDF samples [28]. So, more recrystallized grains appear and grow inside large grains of MC-MDF during solution treatment.

During solution treatment, most of the remaining primary phases and secondary phases dissolved, which reduced obstacles to grain boundary migration [27]. After MDFs, samples of H-MDF have a higher proportion of secondary phases and a lower proportion of primary phases than those of MC-MDF. This is due to the fact that higher forging temperature in the first stage of H-MDF promoted both the dissolution of primary phases and reprecipitation of secondary phases, and the dissolution of primary phases also acted as a source of driven force to the precipitation of secondary phases [34]. Secondary phases were diffused through dislocation diffusion and high-temperature diffusion, then resolved into a matrix during the solid solution process to promote higher aging precipitation-driven force and increase diffusion and number density of aging precipitation phases [30]. With the help of driven force from strain energy, recrystallization grains grown during solution treatment. MC-MDF sample has more CDRX grain within deformed structures with higher strain energy than H-MDF. During solution, most of the deformed structures of MC-MDF with high directionality are transferred into the structure where equiaxial recrystallized grains hybrid distributed with substructure, while samples of H-MDF have limited recrystallized grains located on junction points of grain boundaries. MC-MDF had higher strain energy and GND than that of H-MDF, as presented in Figure 7, which grew the average grain size of MC-MDF from 38.0 µm to 54.5 µm after solid solution through SRX, while that of H-MDF dropped from 54.6 µm to 39.0 µm due to smaller new recrystallized grains being formed. The different trend in grain size change in MDFs during solid solution is mainly caused by different levels of driven force for SRX.

### 4.2. Analysis of Mechanical Properties

During different MDF processes, multiple strengthening mechanisms were applied to the improvement of 7085 aluminum alloy. There were three main types of strengthening mechanisms that contributed to total yield strength ($\sigma_{YS}$): (1) precipitation strengthening ($\sigma_{or}+\sigma_{dis}$); (2) solid solution strengthening ($\sigma_{SS}$); and (3) grain refinement strengthening ($\sigma_{GS}$).

(2) $$\sigma_{YS}=\sigma_{or}+\sigma_{dis}+\sigma_{SS}+\sigma_{GS}$$

Precipitation strengthening in this study was mainly caused by η’ phases within grains. Residual primary phases are too large and less to contribute to strength and could be ignored. The YS of the alloy was highly determined by the size and density of the strengthening phases. From TEM images, precipitated phases of MC-MDF (Figure 8b) at the center had a higher number density but smaller size of phases than that of H-MDF (Figure 8a). Intense lattice distortion and defects like vacancies and dislocations could lead to a higher trend in nucleation during aging treatment. With higher strain energy, more lattice distortion, vacancies, and dislocations formed during the MC-MDF process. The higher density of defects and dislocations contributed to more high-energy areas, which became precipitation nucleating sites during aging treatment and led to a higher number density of precipitating phases. From GND data, samples of MC-MDF presented lower macroscopic dislocation density under the solution state, but a large proportion of GND transformed into microcosmic dislocation and formed more nucleation sites for precipitating during aging.

For the average size of precipitating phases between MC-MDF (8.4 nm) and H-MDF (11.1 nm), the phenomenon was caused by solute concentration differences and dislocation density in the matrix. During the aging process, some unstable and tiny nucleation cores dissolve. There is a critical nucleation radius, where precipitates could grow if larger than this radius, and the size below it would dissolve. The critical nucleation radius r^*^ can be expressed as follows [35]:

(3) $$r^{*}=\frac{2(A+2)\gamma v_{at}}{3kT\ln\frac{C}{C_{eq}}}$$

where *γ* is the interfacial free energy of precipitation and matrix; *v_at_* is atomic volume; *A* is the ratio of the radius to the half-thickness of precipitations; *C_eq_* is the equilibrium solute concentration in the matrix; *C* is the current solute concentration in the matrix; *k* is the Boltzmann constant; *T* is the temperature, K.

The formula above indicates that the critical nucleation radius of precipitates decreased with increasing current solute concentration in the matrix. With higher current solute concentration, the nucleation cores may become more stable, and the precipitates may be easier to exist and grow, increasing the average precipitate size within grains. H-MDF had a higher forging temperature than MC-MDF during the first stage of forging, which dissolved a few more primary phases into the matrix and increased solute concentration. Meanwhile, the higher GND of samples after MC-MDF indicates higher strain energy stored during MC-MDF, which introduced more nucleation sites for aging precipitating. During the aging process, higher nucleation site density caused higher precipitation density and a smaller average size of precipitating phases. This explains why the sample of MC-MDF has a higher density and smaller aging precipitate size. The larger H-MDF precipitate size also left less space for the migration of dislocations and vacancies, making the deformation of grains during the tensile test harder, weakening the strain-hardening effect, and increasing the yield strength than that of MC-MDF.

Previous studies demonstrated that the Orowan dislocation looping mechanism was the main mechanism for precipitates larger than 8 nm [36,37]. However, given that the average sizes of precipitates were 11.1 nm for H-MDF and 8.4 nm for MC-MDF in this work, the contribution from Orowan and strain–field interaction between phase particles and dislocations needs to be considered. For the Orowan model, precipitation strengthening can be described using the following equation [37]:

(4) $$\sigma_{or}=0.84M\left(\frac{Gb}{\lambda }\right)$$

(5) $$\lambda =\left\{\left.{\left(\frac{3\pi }{4f}\right)}^{1/2}-1.64\right\}\right.$$

where *M* = 3.06, presenting Taylor’s factor; *G* = 25.4 GPa for the shear modulus of Al matrix; b = 0.286 nm is Burger’s vector for the FCC crystal; λ indicates the spacing of particles; and *f* and *r* refer to the volume fraction and radius of phase particles.

Thus, the calculated precipitation strengthening contribution from η’ phases and Al_3_Zr of H-MDF was 182.5 MPa, and that of MC-MDF was 133.4 MPa. The difference between the precipitation contribution of H-MDF and MC-MDF is mainly caused by the precipitation growth state, which results in a difference in volume fraction and radius of precipitates. A much higher volume fraction of precipitation in H-MDF contributed more to yield strength than MC-MDF.

$\sigma_{dis}$ represents the interaction effects between phase particles and dislocations due to the coherent relationship, which can be described as follows [38]:

(6) $$\sigma_{dis}=MaGb\rho^{1/2}$$

where a=0.35 is a constant, and ρ is the dislocation density presented by GND. The $\sigma_{dis}$ contribution was 43.3 MPa for H-MDF and 37.3 MPa for MC-MDF, respectively.

Solid solution strengthening occurred when shear modulus and dimensional differences between matrix and solutes hindered dislocation slip. The solid solution effect can be expressed as follows [39]:

(7) $$\sigma_{SS}=MGb\varepsilon_{SS}^{3/2}\sqrt{c}$$

where $\varepsilon_{SS}$ = 0.38 as a constant, and *c* is the solute concentration (wt.%). For the 7085 alloys, there was no interaction between solute elements, and solid solution strengthening introduced by Zr could be ignored for low solubility. The yield strength contribution from Zn, Mg, and Cu were 5.4, 18.6, and 13.8 MPa wt.%^−1^, respectively [40]. The estimated contribution of solution strengthening was around 90.8 MPa in total. However, the main alloying elements were nearly consumed by forming precipitates during aging. Only a few of them remained in the matrix and residual primary phases. H-MDF exhibited a higher contribution to a solution than MC-MDF.

Furthermore, the effect of fine grain strengthening can be expressed by the classic Hell–Petch formula [41]:

(8) $$\sigma_{GS}=\sigma_{0}+\frac{k}{\sqrt{d}}$$

where $\sigma_{0}=$ 20 MPa is the friction resistance to overcome in pure Al during the dislocation migration process; *k* is a constant determined by the properties of the material itself, which is about 0.04 MPa*m^1/2^ for 7085 aluminum alloy; *d* (µm) represents the average grain size; $\sigma_{y}$ (MPa) is the increment in yield strength.

The above formula indicates that the smaller the average grain size, the higher the yield strength of the forging. After solid solution treatment, the average grain size of MC-MDF and H-MDF at CH are 54.5 µm and 39.0 µm, respectively, and the corresponding yield strength contributions from them are 25.4 MPa and 26.4 MPa, indicating that grain size difference only contributes around 1.0 MPa difference on YS based on calculation.

The strain hardening difference was presented on all specimens of H-MDF and MC-MDF based on Figure 4, which were mainly linked to precipitation differences in specimens. From the TEM images of Figure 9, H-MDF samples are calculated to have higher volume fractions of precipitated phases than that of MC-MDF. Meanwhile, from the aging state, the GND values of specimens are 0.31 × 10^14^/m^2^ and 0.20 × 10^14^/m^2^ for H-MDF and MC-MDF, respectively. It is widely known that the strain-hardening effects of aluminum alloys are highly related to precipitation and dislocation density. Usually, the higher the dislocation density is, the less the strain-hardening effect will be shown on the alloy. The same is true for precipitation; the higher the volume fraction of precipitated phases in alloys, the less room for defects and dislocations to move, and the less strain-hardening there will be. Both factors weaken the strain-hardening effects on specimens of H-MDF and explain the lower and earlier yield strength of MC-MDF specimens in all directions.

**Figure 12:**
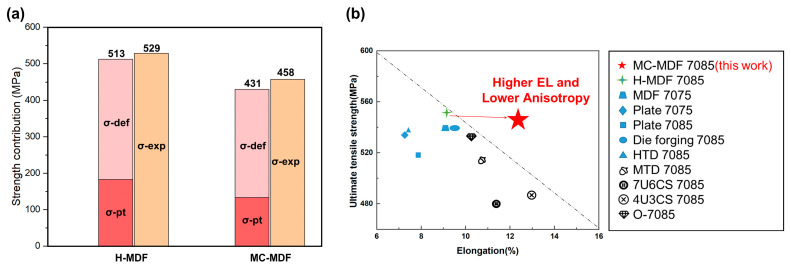
(**a**) Strengthening contributions to YS of H-MDF and MC-MDF, where $\sigma_{pt}$ were calculated values from precipitates, and $\sigma_{def}$ were tensile experiment values from deformed samples; (**b**) corresponding tensile properties of the aged H-MDF and MC-MDF 7085 samples with other existing 7085 and high strength 7xxx alloys [42,43,44,45] fabricated through various MDFs, rolling and die forgings.

To sum up, the strengthening contribution of different sections in H-MDF and MC-MDF samples are presented in Figure 12 and compared the tensile properties of H-MDF, MC-MDF, and others reported 7085 or 7075 alloys which were manufactured through different methods. The results show a good combination of strength and toughness of the MC-MDFed 7085 alloy. The average YS of aged H-MDF samples is 71.0 MPa higher than that of MC-MDF samples. However, the calculated difference between H-MDF and MC-MDF samples was 57.5 MPa, which partially contributed to a higher concentration of solute in H-MDF samples, as presented above. The calculated strength was lower than the measured values, which meant other strengthening mechanisms also existed in alloys, and hard to calculate all strengthening effects [37]. It could be concluded that grain boundary, dislocation interaction, and precipitation strengthening were the primary strengthening modes for both H-MDF and MC-MDF. Also, for every dimension of both forgings, the YS difference between H-MDF and MC-MDF ranged from 40 MPa to 70 MPa, which matched the trend in contribution from precipitation strengthening. The main difference in YS between H-MDF and MC-MDF was mainly caused by aging precipitation differences. And elongation increases in the X and Y directions, as calculated in the results, were likely contributed by decreasing YS in the MC-MDF samples. Even with the loss of YS from H-MDF to MC-MDF, the UTS of MC-MDF maintained the same level on three dimensions; Figure 12b illustrates the excellent strength–ductility synergy of the MC-MDF sample. The delayed work-hardening effect did not cause ultimate strength loss, which was maintained, and increased the industrial application value of MC-MDF.

### 4.3. Relationship Between Microstructure, Mechanical Properties, and Anisotropy

For overall toughness, multiple resources contributed to the EL of MC-MDF than that of H-MDF. After T74 aging, the PFZ width of samples through H-MDF was larger than that of MC-MDF, which decreased grain boundary strength and became a preferential source of failure during the tensile test [31]. Meanwhile, crystalline phases on grain boundaries became stress concentration points, which hindered the movement of dislocations and boundaries and made it easier to fail than grains. For samples of MC-MDF, the width of PFZs was thinner than that of H-MDF, which made MC-MDF harder to extend cracks along GBs. By contrast, H-MDF had larger intracrystalline precipitated phases during aging, which left less space for dislocations and vacancies to move during the tensile test. Cracks happened earlier than that of MC-MDF. Moreover, from SEM images of samples after MDFs (Figure 6a,b), large primary phases are covered by recrystallized grains or sub-grain structures in samples of MC-MDF, while those of H-MDF are still distributed along GBs. Large primary phases can become harder stress concentration points than grain boundaries, which store higher energy, and the recrystallized grains and sub-grain structures grow to enclose those coarse particles. The migration of large particles from GBs to inside grains dominated the trans-granular fracture mode of MC-MDF through higher stress concentration at large particles inside grains. On the other hand, lower YS of samples via MC-MDF than those proceeded by H-MDF indicated that samples of MC-MDF could bear more deformation as a lower fraction of precipitation left more space for dislocations and defects to move, which increased EL. The average increase in EL from precipitation was 9.1% when compared to the EL of H-MDF samples. As a result, MC-MDF showed higher overall toughness than H-MDF, especially in height direction.

MC-MDF had higher EL than that of H-MDF in all dimensions but especially in height direction (12.2% to 9.9%). On the one hand, grain size distribution and grain structures of MC-MDF were more uniform than that of H-MDF, as observed in Figure 6. Narrower size distribution and uniform grain structures could make GBs more uneven, and adjacent grains had similar strength and shape to avoid stress concentration, which resulted in harder extension of crack lines between grains. H-MDF had large grains with a smaller fraction of smaller recrystallized grains mixed in grain structures. Large, deformed structures provided long GBs, and smaller recrystallized grains and large precipitation on GBs supplied stress concentration points. Both effects made GBs weak in samples of H-MDF on height direction, and their cracks presented a higher trend in intergranular attack.

To sum up, the average EL of forging shows the trend that MC-MDF > H-MDF, which is mainly affected by the uniformity of grain structures and the situation of precipitation. The 7085 alloys that were processed via MC-MDF under T74 aging in this work exhibited a combination of good UTS and EL, which was superior to that of H-MDF and most of the reported 7085 alloys. This indicates that the value of MC-MDF supplied a relatively convenient way to acquire high UTS and EL with low anisotropy of forgings.

## 5. Conclusions

(1) At a lower forging temperature of 340 °C during the first forging stage of MC-MDF, CDRX is enhanced due to the increased storage of strain energy, which serves as the driving force for subsequent SRX during the inter-annealing and solution treatment. Due to the higher degree of recrystallization contributed by DRX and SRX, MC-MDFed samples ultimately show highly recrystallized structures across all dimensions. A significant portion of the deformed structures are replaced by recrystallized grains, and the long, straight GBs in the S-T direction are substituted with short, flexural, and new recrystallized GBs. MC-MDF introduces greater deformation into the center of the forgings, which not only increases the recrystallization degree of the center but also ensures a more uniform microstructure of the forgings. Thus, from H-MDF to MC-MDF samples, the EL along the S-T direction increases dramatically, which nearly eliminates the anisotropy of MC-MDFed samples in three dimensions and results in a relatively higher uniformity.
(2) On one hand, H-MDF dissolved more primary phases and increased the solute concentration to enhance the growth of precipitating phases during aging, while MC-MDF retained higher strain energy, which produced more high-energy defects like dislocations and vacancies in samples. These high-energy areas become preferred nucleation sites for precipitation during aging. As a result, the aging precipitates in MC-MDFed samples exhibit a higher number density but a smaller average size. Through calculations, the main differences in YS between H-MDF and MC-MDF are attributed to different degrees of precipitation-strengthening and strain-hardening effects. MC-MDF has a smaller precipitation size and a lower fraction of precipitated phases, which causes an average YS, 54.0 MPa lower than that of H-MDF samples. The lower YS values of MC-MDF samples also contribute to a 9.1% average increase in EL across all dimensions.
(3) After T74 aging, the forging of MC-MDF shows remarkably low anisotropy and excellent overall uniformity, with relatively high mechanical properties when compared to traditional 7085 forgings. Especially in the height direction of MC-MDF samples, the UTS, YS, and EL at the center are 548 MPa, 458 MPa, and 12.2%, respectively. The IDA value of EL decreased from 14.0% of H-MDF to 8.6% of MC-MDF, respectively, accompanied by a more than 20% EL increase in the S-T direction and a 30% IDA decrease. In contrast to existing research, MC-MDF did not exchange ultimate tensile strength for anisotropy but demonstrated a synergy of strength, toughness, and isotropy.

## Figures and Tables

**Table 1:** Mechanical properties of H-MDF and MC-MDF in three directions after T74 aging.

Forging Technology	Mechanical Properties	L-T	T-L	S-T
H-MDF	UTS (MPa)	556 ± 1.0	556 ± 6.5	556 ± 3.2
	YS (MPa)	530 ± 2.9	533 ± 6.1	529 ± 1.4
	EL (%)	12.9 ± 0.49	12.3 ± 0.41	9.9 ± 0.42
MC-MDF	UTS (MPa)	563 ± 2.2	562 ± 3.8	548 ± 3.3
	YS (MPa)	486 ± 2.5	481 ± 8.7	458 ± 0.7
	EL (%)	13.4 ± 0.20	14.0 ± 0.87	12.2 ± 0.02

**Table 2:** IDA value of mechanical properties for the forging deformed via different MDFs.

IDA Value of Mechanical Properties	H-MDF	MC-MDF
UTS	0.0%	1.4%
YS	0.7%	3.4%
El	14.0%	8.6%

## Data Availability

The data presented in this study are available on request from the corresponding author due to privacy restrictions.
